# Management of Psychosis in a Patient with Probable Dopa-Responsive Dystonia

**DOI:** 10.1155/2018/8040491

**Published:** 2018-06-14

**Authors:** Maggie Wang, Joseph I. Sison

**Affiliations:** ^1^University of California Davis School of Medicine, USA; ^2^University of California Davis Department of Psychiatry and Behavioral Sciences, USA

## Abstract

Dopa-responsive dystonia is a rare childhood neurological disorder characterized by asymmetric dystonia, predominantly of the lower limb, that responds excellently to levodopa replacement therapy. Although it is known that behavioral changes, such as depression, anxiety disorders, and sleep disturbances, typically follow onset of motor symptoms, there is limited literature on the psychiatric symptoms of this disorder. This report describes a novel case of a 20-year-old male with a history of dopa-responsive dystonia and schizoaffective disorder who presented with both dystonia and psychosis after a period of medication noncompliance. This case provides a reference for the management of psychosis in patients with dopa-responsive dystonia and highlights the need for more research on the nonmotor symptoms that accompany this neurological disorder.

## 1. Introduction

Dopa-responsive dystonia is a rare condition characterized by childhood onset of limb dystonia (primarily of the lower limbs) that typically generalizes overtime, fluctuate diurnally, and improves with levodopa treatment [1, 2]. It is most commonly caused by an autosomal dominant GTP cyclohydrolase 1 (GCH1) gene defect, but deficiencies in tyrosine hydroxylase, sepiapterin reductase, or other enzymes involved in the biosynthesis of dopamine can also present similarly [1].

More recently, attention has been drawn to an increased frequency of psychiatric symptoms in patients with dopa-responsive dystonia. In multiple studies involving patients with proven GCH1 gene mutation, it was found that they had more psychiatric comorbidities and sleep disturbances compared to the general population [3–5]. However, there have been no documented cases of dopa-responsive dystonia and schizoaffective disorder occurring together.

We present the first case report of a young man with dopa-responsive dystonia and schizoaffective disorder. Although the management of this patient was challenging given that the treatments for these two conditions have counteracting mechanism of actions, we successfully treated his psychotic symptoms with a slow titration of risperidone.

## 2. Case Presentation

A 20-year-old African American man was admitted to a psychiatric facility for psychosis. On initial presentation, the patient had an antalgic gait, which he attributed to his history of dopa-responsive dystonia. His mood was depressed and his affect was restricted. He had disorganized thought process and was slow to recall. He endorsed auditory hallucinations, paranoid delusions, depressive symptoms, frequent night awakenings, and persecutory nightmares. Per the ambulance report, the patient was wandering the streets in a confused state, so bystanders called 911. The patient stated that he had been homeless for the past 3 weeks. During this 3-week period, he admitted to not being complaint with his medications. Urine toxicology screen was negative.

Per medical records, he was diagnosed with dopa-responsive dystonia at age 11 after a 2.5-year history of progressive abnormal gait. He was initially misdiagnosed with tight heel cords at age 10 and treated with serial casting that resulted in good improvement on the right but marginal improvement on the left. His toe walking became more pronounced overtime accompanied by worsening left calf pain and stiffness, increasingly frequent falls, and new onset of intermittent torticollis. These symptoms worsened over the course of the day. He was eventually taken to an urban teaching hospital, where he was diagnosed with dopa-responsive dystonia based on clinical presentation and marked improvement on a levodopa trial. Magnetic resonance imaging of the brain and spine was unremarkable at the time.

At age 15, he was diagnosed with schizoaffective disorder bipolar type. His psychiatric history is also significant for multiple psychiatric hospitalizations, history of previous suicide attempts with medication overdose, and history of trauma. He also endorsed marijuana use since age 15 and daily tobacco use since age 18. He denies using any other illicit drugs. Per collateral information from his mother, his schizoaffective disorder has never been well controlled given the conflicting effects of his medications. She also mentioned that he was placed in individualized education programs as a child due to learning disabilities. His family history is significant for bipolar disorder on his maternal side. His family history on his paternal side is unknown. In addition to carbidopa-levodopa, his outpatient medications included sertraline, divalproex sodium, aripiprazole, and benztropine.

On hospital day 1, he was started on carbidopa-levodopa 25/100 mg tablet three times daily for dopa-responsive dystonia. On day 2 of his hospital course, sertraline 50 mg once daily, benztropine 2 mg twice daily, divalproex sodium 500 mg twice daily, and risperidone 0.5 mg twice daily were added to his medication regimen. We started him on a low-dose risperidone to avoid exacerbating his dopa-responsive dystonia symptoms. Physical exams were also performed daily to assess for dystonia and parkinsonian symptoms. His initial physical exam revealed an antalgic gait secondary to left lower extremity dystonia, which improved by hospital day 2 and resolved by hospital day 3. On hospital day 3, he became agitated and aggressive with staff members, which led to intramuscular administrations of haloperidol 10 mg, diphenhydramine 50 mg, and lorazepam 2 mg. He continued to endorse auditory hallucinations, so risperidone was increased to 0.5 mg in the morning and 1 mg at bedtime. His auditory hallucinations resolved and then returned on day 6. He reported hearing “good” voices and “bad” voices. He also continued to endorse depressive symptoms, multiple night awakenings, and persecutory nightmares. As a result, his risperidone dosage was increased to 1 mg twice daily. On hospital day 7, the patient reported hearing “mumbling” voices only and improvement in his sleep and depressive symptoms. On hospital day 8, his auditory hallucinations fully abated. By hospital day 10, he slept throughout the night, no longer had depressive symptoms, and had normal spontaneous speech. His thought process was linear, logical, and goal-oriented. His mood and affect was euthymic and full range. No psychotic symptoms were noted. The patient was compliant with his medications throughout the whole hospital course and his daily physical exams were negative for dystonia or parkinsonian symptoms since day 3 of his hospitalization. He was subsequently discharged on hospital day 14 with appropriate outpatient follow-up.

## 3. Discussion

Dopa-responsive dystonia is a rare childhood neurological disorder that typically starts as asymmetric lower limb dystonia, resulting in equinovarus foot posturing and gait disturbances [2]. These symptoms worsen in the evening, improves in the morning after sleep, and resolves with levodopa treatment. The most extensively studied cause of dopa-responsive dystonia is Segawa Disease, which is due to an autosomal dominant mutation in the GTP cyclohydrolase 1 (GCH1) gene [1]. This genetic defect results in a deficiency of GCH1. GCH1 is an enzyme that mediates the rate-limiting step in the biosynthesis of tetrahydrobiopterin (BH_4_), an essential cofactor in the production of dopamine. Reduction in dopamine interferes with the brain's ability to produce smooth physical movement, resulting in dystonia, tremor, and/or parkinsonian symptoms. Similar presentations can also be seen in autosomal recessive mutation in GCH1 and deficiency in tyrosine hydroxylase, sepiapterin reductase, or other enzymes involved in the biosynthesis of dopamine [1].

Recently, there has been more focus on the psychiatric symptoms of dopa-responsive dystonia. In a study of 18 patients with proven GCH1 gene deficiency, it was found that they had increased frequency of major depressive disorders, anxiety disorder, obsessive-compulsive disorder, and sleep disturbances compared to the general population [3]. In another study involving 34 patients with confirmed mutations in the GCH1 gene, more than half of the cases presented with psychiatric symptoms, including depression and panic attacks. These behavioral changes did not precede motor signs in any of the subjects [4], indicating that psychiatric symptoms are late manifestations of the disease.

The patient described in this case report is unique in that he presented with psychotic symptoms consistent with schizoaffective disorder, which interestingly has never been documented in patients with dopa-responsive dystonia. Although it can be argued that we may have coincidentally observed two separate pathologies occurring together, we also cannot exclude the possibility that his psychotic symptoms were secondary to his neurological disorder. Levodopa-induced psychosis is also under consideration given that it has been shown that levodopa can lead to behavioral changes, including psychosis and mania, in patients with Parkinson's Disease [6–8]. However, in our case, the patient continued to exhibit auditory hallucinations and disorganized thought process during a 3-week period of medication noncompliance, which makes levodopa-induced psychosis less likely since his psychotic symptoms should have resolved with discontinuation of levodopa.

Similar to managing Parkinson's Disease patients with psychotic symptoms, treating both dopa-responsive dystonia and psychosis is difficult because the treatments for these conditions have counteracting mechanisms of actions. On one hand, levodopa replacement therapy increases dopamine levels in the central nervous system, which would exacerbate psychotic symptoms. On the other hand, antipsychotics antagonizes dopamine receptors, worsening motor symptoms in dopa-responsive dystonia. In our case, we chose to restart the patient on carbidopa-levodopa to control his dystonic symptoms before addressing his psychotic episode. Once his motor symptoms were under control, we started him on 0.5 mg of risperidone twice daily for psychosis and 2 mg of benztropine twice daily to prevent extrapyramidal symptoms. One of the reasons why we chose risperidone was because of its linear pharmacokinetics [9, 10]. Although aripiprazole also has a linear dose-response [11], we decided on a different antipsychotic because the patient had responded poorly to aripiprazole in the past. The other reason why we chose risperidone was because it has been shown to be effective at treating psychosis in patients with Parkinson's Disease [12]. Since recent literature has suggested that dopa-responsive dystonia may be associated with Parkinson's Disease [13, 14], we postulated that risperidone would also be effective at treating psychosis in dopa-responsive dystonia.

Over the course of a week, risperidone was slowly titrated up by 0.5 mg at a time, while simultaneously monitoring for extrapyramidal and parkinsonian symptoms. It is necessary to start the antipsychotic at a low dose and slowly titrate up, as starting at a higher dosage or titrating too quickly can potentially exacerbate motor symptoms of dopa-responsive dystonia. Therapeutic effect was eventually reached at 2 mg daily.

The biggest limitation of this case was the lack of genetics testing to confirm his diagnosis of dopa-responsive dystonia. With the patient's permission, we had obtained previous medical records from the children's hospital in which he was diagnosed with dopa-responsive dystonia. His medical records indicated that he had been diagnosed based on clinical presentation only. We speculate that the physicians caring for him at the time did not pursue genetic testing because it would not have changed the management for this patient. It has also been noted that commercially available DNA tests for this condition are not comprehensive [1], so there is a possibility that genetics testing in this case would have not provided us with any new information.

In conclusion, this case demonstrates that risperidone is effective in managing psychotic symptoms in a patient with dopa-responsive dystonia. This case also brings up the question on whether psychosis is a late manifestation of dopa-responsive dystonia. As of date, there is no literature citing psychosis as a nonmotor symptom of dopa-responsive dystonia, but recent studies have noted increased frequency of psychiatric symptoms in patients with dopa-responsive dystonia. Hence, further investigation is needed to elucidate the relationship between psychosis and dopa-responsive dystonia.
